# Why People Share (Or Don’t): Race/Ethnicity and Contextual Correlates of Willingness to Disclose Contact Information During the COVID-19 Pandemic in Rural North Carolina

**DOI:** 10.3390/ijerph23020267

**Published:** 2026-02-20

**Authors:** Leah J. Floyd, Irene Doherty, Tanisha Burford, Deepak Kumar

**Affiliations:** 1 Julius L. Chambers Biomedical and Biotechnology Research Institute, North Carolina Central University, Durham, NC 27707, USA; idoherty@nccu.edu (I.D.); dkumar@nccu.edu (D.K.); 2 Department of Psychology, North Carolina Central University, Durham, NC 27707, USA; tburford@nccu.edu

**Keywords:** COVID-19, contact tracing, rural health, health equity, trust, public health communication, race/ethnicity, access

## Abstract

**Highlights:**

**Public health relevance—how does this work relate to a public health issue?**

- Contact tracing has proven useful, but challenging.
- Given the potential of contact tracing as an effective tool for controlling the spread of communicable diseases, further investigation into factors that contribute to its successful implementation is warranted.

**Public health significance—why is this work of significance to public health?**

- We seek to expand knowledge by examining the influence of race/ethnicity and trust on contact tracing compliance, while controlling for access to health services during a public health crisis in a rural sample in the southern United States.
- A better understanding of the individual and synergistic influences of race/ethnicity, interpersonal and macro-level factors on an individual’s willingness to share information with contact tracers in rural economically distressed areas has implications for improving the effectiveness of contact tracing in low-resourced communities.

**Public health implications—what are the key implications or messages for practitioners, policy makers and/or researchers in public health?**

- Findings support research that suggests contact tracing is a viable strategy for mitigating COVID-19 transmission in rural communities when trust in health care providers is high and access to testing is limited, regardless of race.
- The lack of racial differences in the association between trusting health care providers and engagement in contact tracing suggest the interactive effects of race and trust on engagement in public health interventions may be nuanced and requires that more attention be given to place and time.
- Health care providers and public health officials should work to build strong, trust-based relationships with community members that will, in turn, facilitate adherence to contact tracing recommendations during a crisis.

**Abstract:**

For historically marginalized groups and residents of low-resource rural communities, contact tracing is a critical tool for controlling the spread of communicable diseases. To improve its effectiveness, more research on identifying factors that influence an individual’s willingness to comply with contact tracers is needed. Therefore, we examined the association of race/ethnicity, contextual factors, and willingness to engage in contact tracing during the COVID-19 pandemic. The sample included 337 adults (56% Black/African American and 66% female). Approximately 80% of the participants indicated they would disclose the names of contacts. The results from the multivariate logistic regression analyses indicated lack of access to COVID-19 testing sites (aOR = 2.20; 95% CI = 1.08–4.48) and trust in health care providers (aOR = 7.57; 95% CI = 3.82–14.88) were significantly associated with willingness to share information with contact tracers. Race did not moderate the relationship between trust and engaging with contact tracers. The results suggest contact tracing is a viable strategy for mitigating disease transmission in rural communities, particularly when trust in health care providers is high and access to testing is limited, regardless of race. Public health officials should invest in maintaining contact tracing teams that include medical providers and prioritize building trusting relationships with all community members.

## 1. Introduction

In 2019, the severe acute respiratory syndrome coronavirus 2 (SARS-CoV-2)—the virus that causes COVID-19—emerged in metropolitan areas and quickly spread to rural counties throughout the United States [1,2,3]. As the pandemic progressed, racial/ethnic minorities (e.g., Black/African Americans and Hispanics), and residents of low-resource rural communities experienced disproportionately higher COVID-19 incidence [4,5], disease severity and hospitalization [6,7,8], and death [9,10]. In the United States, 1.23 million individuals have died of COVID-19, to date [11].

### 1.1. Contact Tracing

Presently, COVID-19 no longer poses a significant public health threat because of herd immunity, which is largely attributable to vaccines [12]. However, early in the COVID-19 pandemic, when effective pharmacological options were not available, contact tracing served as a primary strategy for controlling the spread of the disease [13,14,15]. The general process for contact tracing involves identifying, notifying and quarantining individuals who have had close contact with new cases to prevent further transmission within the community [16]. Having been proven to be somewhat effective for reducing the transmission of communicable diseases, contact tracing will continue to be an essential public health tool for mitigating the spread of acute infections with short incubation periods, such as the SARS-CoV-2 [13,14,15,16,17], especially in low-resource rural settings and among marginalized and disadvantaged populations with limited access to primary and tertiary care.

Although contact tracing has been shown to be a viable public health strategy for controlling the spread of communicable diseases, such as COVID-19, its effectiveness varies [15,18]. One factor influencing the effectiveness of contract tracing is the willingness of exposed individuals to share the information of persons with whom they have been in close contact with public health workers (i.e., contact tracers) [18,19]. While willingness to share information is an individual-level behavior, it is influenced by myriad non-medical contextual factors that impact both health behaviors and health outcomes (often referred to as social determinants of health). Factors that can influence health behaviors, such as communicating openly and honestly with public health workers, range from more direct interpersonal factors (e.g., trust) to more distant macro-level contextual factors, such as lack of access to prevention and treatment services [20,21]. Therefore, it is critical that researchers identify factors across the social ecology system that can be modified to improve the implementation of contact tracing.

### 1.2. Trust in Health Care Providers

Trust, or willingness to be vulnerable to another, is a malleable interpersonal factor that directly influences the effectiveness of contact tracing [22,23]. Unfortunately, in most contact tracing situations there is no prior opportunity to build trusting and mutually respectful relationships between the individual public health worker and community members. By and large, the level of trust for the contact tracer is dependent upon the community members’ pre-existing beliefs about their health care provider and the medical system [22,23,24]. For example, Randall et al. reported “trust in contact tracers was associated with increased intentions to comply with tracing requests and significantly mediated the positive relationship between trust in healthcare professionals and government health officials with compliance intentions” 23 (p. 1). This transference of trust is known as swift trust [24].

In health equity research, group differences in the degree to which racial/ethnic groups trust health care professionals and public health agencies have emerged, and these differences have been associated with disparities in a variety of health outcomes [25,26,27,28,29,30,31,32,33]. Within the COVID-19 literature, specifically, racial and ethnic differences in trust in public health and medical personnel are associated with variation in clinical trial participation, vaccine hesitancy, and contact tracing engagement [21,23,27,30,33]. For example, in a survey conducted by the Pew Research Center in 2020, Hispanic adults were less likely to engage in the contact tracing process, compared to other groups [25]. Similarly, Kas-Osoko et al. found that, although African Americans viewed contact tracing as beneficial, medical and government mistrust were major barriers to participating in contract tracing efforts [21]. This lack of trust exhibited by African Americans and Hispanics has been attributed by some scholars to systemic racism and discrimination [31].

### 1.3. Access to COVID-19 Testing Sites

An individual’s willingness to share information may also be influenced by external macro-level provisions. Health care access (e.g., free testing sites) is among the key societal-level contextual factors that have been associated with poor COVID-19 outcomes in rural areas and in economically and socially disadvantaged communities [30,32,33,34]. The availability of free testing sites facilitates prevention by making it easier for individuals to confirm their status. However, the influence of access to testing sites may not be limited to its direct impact on testing behaviors. Limited access to testing services can have a broader impact by influencing related health behaviors, such as contact tracing. In particular, lack of access to COVID-19 testing may heightened perceived susceptibility, as uncertainty regarding infection status and network members’ disease status can heighten individuals’ perceptions of personal risk. This uncertainty may also amplify perceived disease severity by elevating concern about potential health consequences for oneself and one’s family and friends. Within this context, greater willingness to disclose network members’ names to a contact tracer may reflect an urgent, compensatory response to elevated risk perceptions in settings characterized by restricted access to preventive resources. Thus, it is probable that limited access to facilities that offer free testing increases the willingness of community members to adopt other preventive behaviors, such as willingness to engage in contact tracing.

### 1.4. Current Study

Contact tracing has proven useful, but challenging. Given its potential as a tool for controlling the spread of communicable diseases, further investigation into factors that contribute to its successful implementation in under-resourced rural communities is warranted. Therefore, the purpose of this paper is to: (1) examine the degree to which willingness to engage in contact tracing is associated with trusting health care providers, and (2) assess whether the association between trusting health care providers and engaging in contact tracing varies by race/ethnicity, while controlling for sociodemographic factors and access to COVID-19 testing sites—a macro-level social determinant of health. This study addresses several gaps in the literature. First, most of what is known about the relationship between race/ethnicity, trust in health care systems, and health behaviors is not specific to contact tracing or behaviors during a public health crisis. Second, the preponderance of the literature is based on data from urban populations. A better understanding of the individual and synergistic influences of race/ethnicity, interpersonal and macro-level factors on rural individuals’ willingness to share information with contact tracers during a pandemic has implications for improving the effectiveness of contact tracing in rural socially, economically, and medically underserved communities, and may inform future responses to public health emergencies in these settings.

## 2. Materials and Methods

The data for this paper are from the ACCORD STUDY and were collected between August 2020 and December 2020 [35]. Data collection occurred at community events held in eight rural counties in North Carolina. As an HBCU, North Carolina Central University (NCCU) has fostered trusting and collaborative relationships with Black/African American underserved communities throughout North Carolina. The NCCU ACCORD team leveraged their existing relationships with public health departments, community-based organizations and community members in each county to organize COVID-19 testing events. Six of the eight counties were classified as Tier 1 (most economically distressed) by the North Carolina Department of Commerce. Data collection involved trained study staff inviting individuals receiving COVID-19 testing services to complete a brief survey designed to assess health behaviors and contextual factors related to COVID-19 transmission. The survey included questions designed to measure demographic factors, socioeconomic status, contact tracing compliance, and barriers to COVID-19 testing. The web-based survey required approximately 10 min to complete. The only study eligibility criterion was being 18 years of age or older. No incentive was provided. The NCCU Institutional Review Board approved the study.

### 2.1. Measures

*Sociodemographic measures*: Participants reported their race and ethnicity, age, sex, and their highest level of education.

*Financial strain*: What bills have you had trouble paying due to COVID-19? (Check all that apply). Ten response options were included ranging from (10) mortgage/rent to (0) prefer not to answer. Two groups were created (financial strain and no financial strain) If participants endorsed any difficulty paying a bill, they were assigned to the financial strain group.

*Trust*: Who would you trust to provide you with information about contact tracing?

Response options included (1) health department, (2) health care provider, (3) community service agency, (4) someone you relate to, and (5) pastor/faith leader. Participants were asked to select all that apply.

*Access to testing*: What would or has already prevented you from getting tested? (Check all that apply.) Respondents could choose from 10 response options including (1) no place to get tested in your community and (2) don’t know where to go to get tested. Responses to these two items were combined to create the access to testing variable.

*Contact tracing*. Sometimes people who get infected may not want to tell a contact tracer about all of the people in their network of contacts. Would you feel comfortable providing contact tracers with information to contact…? Response choices included (1) none of them, (2) some but not all of them, and (3) all of them. For data analytic purposes, we created two groups, a willingness-to-disclose group and a non-disclosure group. Respondents who selected willingness to provide information for some or all their contacts were classified as “willing to disclose”.

### 2.2. Analysis

We began by examining each variable for missingness. Age was the only variable with more than 3% of data missing at random. To replace the missing values, we used the series mean method in SPSS. Next, we generated descriptive statistics and employed the Chi square test to examine differences across race/ethnicity for the main variables. Logistic regression analyses were employed to identify correlates of willingness to disclose the names of contacts. We report odds ratio (OR) and adjusted odds ratio (aOR) with their 95% confidence intervals (CIs). All analyses were conducted using SPSS 29 (IBM Corp, 2022).

## 3. Results

### 3.1. Descriptive Statistics

The current paper includes 337 individuals who identified as Black/African American, Hispanic or White. Participants who did not identify as a member of one of the aforementioned groups (*N* = 20) were excluded from the analyses. The mean age of the sample was 51.2 (SD = 6.98) years. Black/African Americans comprised 56% of the sample, 30% of participants self-identified as white and 14% as Hispanic. Females were 66% of the sample. Approximately 64% of the respondents indicated they trusted their health care provider to provide information about contact tracing, and 80% indicated they would participate in contact tracing efforts. Most of the sample reported no access to COVID-19 testing (77%). Twenty-three percent of Hispanic participants, compared to 70% of Black/African American participants and 74% of white participants, reported trusting their health care provider to provide them with information about contact tracing. Perceived lack of access to COVID-19 testing sites varied by race/ethnicity (77%, 65%, and 85% for Black/African Americans, Hispanics, and white participants, respectively). See Table 1 for a detailed summary of the sample characteristics.

### 3.2. Logistic Regression Analyses

Results from the bivariate logistic regression analysis indicated a significant positive relationship between trusting a health care provider for information about contact tracing and willingness to disclose the names of exposed network members to a contact tracer (OR = 6.94; 95% CI = 3.83–12.61). A significant relationship between willingness to disclose network members’ names to a contact tracer and lack of access to COVID-19 testing sites emerged (OR = 3.32; 95% CI = 1.86–5.91). Race/ethnicity was not associated with willingness to disclose contact information. 

Next, to test the full model we employed hierarchical logistic regression analysis that included all covariates, in addition to the race/ethnicity by trust interaction term. In the first step, age, race/ethnicity, sex, education, financial strain, access to testing, and trust, were entered into the equation as covariates with contact tracing as the outcome. Two significant associations emerged. Trust in health care providers was significantly associated with willingness to disclose information to a contact tracer (aOR = 7.57; 95% CI = 3.82–14.88). Lack of access to a COVID-19 testing sites was significantly associated with engaging in contact tracing (aOR = 2.20; 95% CI = 1.08–4.48). In Step 2, the race/ethnicity by trust interaction term was introduced into the model. The race/ethnicity by trust interaction for African Americans and white Americans was not statistically significant (aOR = 0.72; 95% CI = 0.16–3.24). Due to the small number of Hispanic Americans, the race/ethnicity by trust interaction term that compared Hispanic Americans and white Americans could not be adequately tested. The final model accounted for 24% of the variance in contact tracing (Nagelkerke R^2^ = 0.24). Table 2 provides a detailed summary of the the resu;ts from the logistic regression model.

## 4. Discussion

During the COVID-19 pandemic, contact tracing reduced the transmission of SARS-Cov-2 and undoubtedly saved lives [13,14,15]. Although effective, successful implementation of contact tracing is often described as challenging. Consequently, there remains a need for research to delineate factors that influence an individual’s willingness to comply with contact tracers. Therefore, we examined the extent to which race and ethnicity, trust in health care providers, and access to COVID-19 testing were associated with an individual’s willingness to comply with contact tracers in a rural sample. The expected racial/ethnic differences in willingness to share names and personal information of network members with contact tracers did not emerge. Similarly, differences in trust in health care providers to share information about contact tracing for Black/African American and white participants were not evident. However, compared to Black/African American and white participants, a smaller percentage of Hispanic participants reported trusting health care providers. But given the small number of Hispanic participants in the study, this finding should be interpreted with caution. Lastly, a significant relationship between trust in health care providers and willingness to participate in contact tracing emerged. Individuals who trusted their health care provider to share information about contact tracing were seven times as likely to report a willingness to engage in contact tracing efforts, compared to those who did not trust their health care provider to provide information about contact tracing. This relationship did not differ for Black/African American and white participants. Due to the small number of Hispanic participants, the interaction term including Hispanic and white participants could not be adequately tested.

Interestingly, we did not find the expected race-based differences in trust in health care providers and participation in contact tracing among Black/African American and white participants. Although it is frequently argued that Blacks/African Americans have higher levels of distrust for medical and government institutions, recent empirical findings supporting this claim are inconsistent [23,25,27,36]. For example, similarly to our study findings, Randall et al. found urban African American and white adults reported comparable levels of trust in health care providers and intention to comply with contact tracers during the first year of the COVID-19 pandemic [23]. Moreover, ecological models and analyses situated in a health equity framework are increasingly demonstrating that race/ethnicity—once promoted as a biological construct—is a proxy for a complex interaction of environmental, economic, social, and cultural factors [37,38]. The lack of racial differences in the association between trusting health care providers and engagement in contact tracing suggest the moderating effects of race on the relationship between trusting a health care provider and engagement in public health interventions is nuanced and requires that more attention be given to place and time. Therefore, broadly applying the underpinnings of African Americans’ distrust of government, medicine, and public health as a barrier for ameliorating health disparities should be avoided, as this likely blurs important subtleties that could inform practice and policy [29,36].

Furthermore, a more comprehensive understanding of the lack of racial/ethnic differences in levels of trust requires consideration of the unique circumstances surrounding the pandemic. The COVID-19 pandemic was an unprecedented time of uncertainty, fear, and rapidly evolving public health guidance, along with widespread misinformation. Given the novelty and severity of COVID-19, the extent to which distrust, rooted in historical and contemporary trauma and discrimination, influenced decision-making about cooperating with contact tracers may have been reduced during the pandemic. Thus, racial/ethnic differences in the influence of trust on engaging in contact tracing may have been less salient during the COVID-19 pandemic than what has been observed for other health outcomes in previous years. However, more empirical evidence is required to confirm this pattern. Nonetheless, considering research that suggests trusting relationships with health care providers enhance individuals’ willingness to share contact information [19], scaling up evidence-based interventions that strengthen providers’ capacity to deliver high-quality, compassionate, and unbiased care is essential. In addition, health care providers should be trained in contact tracing. Such efforts will likely improve health outcomes across populations and represent an important step toward achieving health equity.

Lastly, consistent with other reports of COVID-19 testing shortages [39,40,41], the majority of study participants (three out of four) reported no testing sites in their community. In our study, limited access to COVID-19 testing sites also emerged as an important contextual factor related to willingness to engage in contact tracing. Specifically, individuals who reported no access to a COVID-19 testing site were twice as likely to participate in contact tracing, compared to their counterparts who reported access to a testing site. It is logical to argue that during the COVID-19 pandemic the lack of access to testing services impelled the uptake of other preventive programs and behaviors, such as participation in contact tracing. Lack of access to COVID-19 testing may function as a proxy for heightened perceived susceptibility. Although access to testing was not a direct measure of perceived susceptibility, our findings may signal that environmental constraints could indirectly shape engagement and cooperation with public health interventions (e.g., contact tracing) by amplifying perceived risk and urgency.

While the study’s findings are significant, the limitations of this study must be noted. One notable limitation is the recruitment strategy employed. The use of a non-probability venue-based sample limits our ability to generalize the study findings to all persons residing rural counties in North Carolina. Employing random sampling techniques would yield a more representative sample and improve external validity. In addition, future studies may benefit from a larger sample size to increase the power to employ more rigorous multi-level analyses capable of adequately testing interactions and quantifying the influence of individual and community level effects. Doing so will improve predictive validity.

## 5. Conclusions

In conclusion, the findings support the limited research that suggests contact tracing is a viable strategy for mitigating COVID-19 transmission in rural communities in North Carolina when trust in health care providers is high and access to testing is limited, regardless of race/ethnicity. Therefore, health care providers and public health officials should prioritize building strong, trust-based relationships with community members that will, in turn, facilitate adherence to contact tracing in critical times. Investment in the development of well-trained contact tracing teams that include health care providers should also be prioritized. Additional research is warranted.

## Figures and Tables

**Table 1:** Sample Characteristics.

Variable	Total N	%	African American N	%	Hispanic N	%	White N	%	X^2^ (*p*-Value)
Sex * Female Male	222 112	66.5 33.5	129 57	69.4 30.6	37 11	77.1 22.9	56 44	56 44	8.0 (0.018)
Education * No high school diploma High school diploma Some college/trade Bachelor’s degree	47 104 85 88	14.2 32.1 26.2 27.2	27 70 51 38	14.5 37.6 27.4 20.4	11 9 18 11	28.2 23.1 20.5 28.2	9 25 26 39	9.1 25.3 26.3 39.4	20.5 (0.002)
Financial strain	125	37.1	77	40.7	24	50.0	24	24	11.8 (0.003)
No access to testing	261	77.4	145	76.7	31	64.6	85	85	7.9 (0.02)
Trust health care provider	217	64.4	132	69.8	11	22.9	74	74	42.5 (<0.001)
Contact tracing compliance	270	80.1	148	78.3	37	77.1	85	85	2.2 (0.339)

* Missing data or prefer not to answer.

**Table 2:** Results of logistic regression analyses examining the association of race/ethnicity, contextual factors and willingness to participate in contact tracing.

Variable	N	%	Unadjusted Odds Ratio	95% CI	Adjusted Odds Ratio	95% CI
Age	-		1.0	1.00, 1.02	1.0	0.98, 1.02
Race/Ethnicity African American Hispanic White	148 37 85	78.3 77.1 85	0.64 0.59 1.0	0.33, 1.22 0.25, 1.41 --	0.65 1.59 1.0	0.30, 1.41 0.52, 4.90 --
Sex Female Male	176 93	79.3 83	1.0 1.28	-- 0.71, 2.31	1.0 1.66	0.82, 3.37
Education No High School Diploma High School Diploma Some College/Trade Bachelor’s Degree	39 83 70 70	83 79.8 82.4 79.5	1.25 1.02 1.20 1.0	0.50, 3.15 0.50, 2.06 0.56, 2.57 --	1.78 1.71 1.17 1.0	0.62, 5.11 0.74, 3.93 0.49, 2.79 --
Financial Strain Yes No	102 168	81.6 79.2	1.16 1.0	0.66, 2.04 --	1.42 1.0	0.72, 2.80 --
Access to Testing Yes No	222 48	85.1 63.2	1.0 3.32	-- 1.86, 5.91	1.0 2.20	-- 1.08, 4.48 *
Trust Health Care Provider Yes No	198 72	91.2 60	6.94 1.0	3.83, 12.61 --	7.57 1.0	3.82, 14.88 **

* *p* < 0.05, ** *p* < 0.001.

## Data Availability

Data are unavailable due to privacy restrictions. Because the quantitative data include detailed contextual information that cannot be fully de-identified without significantly compromising their integrity, data sharing would not meet the standards required to ensure participant confidentiality.
